# Methamphetamine induces transcriptional changes in cultured HIV-infected mature monocytes that may contribute to HIV neuropathogenesis

**DOI:** 10.3389/fimmu.2022.952183

**Published:** 2022-08-18

**Authors:** Vanessa Chilunda, Jessica Weiselberg, Samuel Martinez-Meza, Lwidiko E. Mhamilawa, Laura Cheney, Joan W. Berman

**Affiliations:** ^1^ Department of Pathology, Albert Einstein College of Medicine, Bronx, NY, United States; ^2^ Department of Parasitology and Medical Entomology, Muhimbili University of Health and Allied Sciences, Dar es Salaam, Tanzania; ^3^ Department of Women’s and Children’s Health, International Maternal and Child Health (IMCH), Uppsala University, Uppsala, Sweden; ^4^ Department of Medicine, Division of Infectious Diseases, Montefiore Medical Center and Albert Einstein College of Medicine, Bronx, NY, United States; ^5^ Department of Microbiology and Immunology, Albert Einstein College of Medicine, Bronx, NY, United States

**Keywords:** methamphetamine, monocytes, HIV, neuroinflammation, migration, viral reservoirs

## Abstract

HIV-associated neurocognitive impairment (HIV-NCI) persists in 15-40% of people with HIV (PWH) despite effective antiretroviral therapy. HIV-NCI significantly impacts quality of life, and there is currently no effective treatment for it. The development of HIV-NCI is complex and is mediated, in part, by the entry of HIV-infected mature monocytes into the central nervous system (CNS). Once in the CNS, these cells release inflammatory mediators that lead to neuroinflammation, and subsequent neuronal damage. Infected monocytes may infect other CNS cells as well as differentiate into macrophages, thus contributing to viral reservoirs and chronic neuroinflammation. Substance use disorders in PWH, including the use of methamphetamine (meth), can exacerbate HIV neuropathogenesis. We characterized the effects of meth on the transcriptional profile of HIV-infected mature monocytes using RNA-sequencing. We found that meth mediated an upregulation of gene transcripts related to viral infection, cell adhesion, cytoskeletal arrangement, and extracellular matrix remodeling. We also identified downregulation of several gene transcripts involved in pathogen recognition, antigen presentation, and oxidative phosphorylation pathways. These transcriptomic changes suggest that meth increases the infiltration of mature monocytes that have a migratory phenotype into the CNS, contributing to dysregulated inflammatory responses and viral reservoir establishment and persistence, both of which contribute to neuronal damage. Overall, our results highlight potential molecules that may be targeted for therapy to limit the effects of meth on HIV neuropathogenesis.

## Introduction

HIV remains a major public health issue, with approximately 38 million people worldwide currently living with the virus (1). Antiretroviral therapy (ART) has dramatically improved the lifespan of people with HIV (PWH). However, a significant percentage of PWH develop comorbidities that significantly impact their quality of life. One such comorbidity is HIV-associated neurocognitive impairment (HIV-NCI), which occurs in about 15-40% of PWH despite suppressed viremia (2–8). HIV-NCI is a spectrum of disorders that can fluctuate over time and includes, but is not limited to, impaired executive function, working memory, and attention (2). People with HIV-NCI have a premature decline in overall physical and psychological health, with difficulties maintaining employment and social relationships and in performing instrumental activities of daily living (9–13). HIV-NCI is an independent risk factor for mortality, and currently, there are no effective treatments in the context of ART (14–16). The development of HIV-NCI is complex, resulting from both viral seeding and reseeding, and chronic inflammation and neuronal damage to the central nervous system (CNS).

Substance use disorder (SUD), another highly significant public health issue, also contributes to HIV-NCI in PWH. In a large cohort study, SUD was reported in approximately 48% of PWH, with 13% of those using methamphetamine (meth) (17). Meth is a stimulant that readily crosses the blood-brain barrier (BBB), and can cause neuronal damage directly, and indirectly by affecting microglia and astrocytes that provide neuronal support (18). People without HIV who use meth chronically have been shown to have increased neuronal injury and neurocognitive impairment compared to those who do not use meth (19–21). Studies that examined the impact of meth on neuronal health in PWH showed that those who use meth have decreased neuronal health compared to people without HIV who use meth and compared to PWH who do not use meth (22, 23). Other studies have shown that PWH who use meth have increased neurocognitive impairment compared to the other groups (24, 25). These data suggest that meth may exacerbate HIV neuropathogenesis in PWH.

HIV entry into the CNS, which is critical to development of HIV-NCI, occurs, in part, by the transmigration of HIV-infected monocytes across the BBB (26–30). These infected monocytes produce virus that can infect and activate resident CNS cells (31, 32). This results in production of host and viral factors that are neurotoxic and can also recruit additional monocytes from the peripheral blood into the CNS. Intermediate monocytes, a specific subset of monocytes that expresses surface CD14, the LPS co-receptor, and CD16, the FcγRIII receptor, are key mediators of this process (27). The CD14^+^CD16^+^ intermediate monocytes are referred to as mature monocytes throughout the text. These cells are preferentially infected with HIV and preferentially transmigrate across a BBB model compared to other monocyte subsets (28, 33, 34). These monocytes are increased in the peripheral blood of PWH and they are increased further in those who use substances, including meth, demonstrating that meth can affect these monocytes (35–37). The infected monocytes can differentiate into long-lived macrophages that help sustain viral persistence, neuroinflammation, and neuronal damage. *In vivo* studies showed that meth increased the population of CD14^+^CD16^+^ macrophages and the amount of virus in the brains of Simian Immunodeficiency Virus (SIV) infected macaques (38, 39). PWH who use meth were shown to have increased plasma and cerebrospinal fluid (CSF) viral loads despite adherence to ART (23, 40, 41). These studies suggest that meth may increase viral replication as well as the entry of HIV-infected mature monocytes into the CNS, thus contributing to viral seeding/reseeding and exacerbated neuroinflammation and subsequent neuronal damage.

The effects of meth on peripheral HIV-infected mature monocytes that can contribute to HIV neuropathogenesis in PWH have not been extensively characterized. In this study, we performed RNA sequencing of HIV-infected mature monocytes treated with or without meth. We demonstrate that meth treatment of HIV-infected mature monocytes increases expression of transcripts related to cytoskeletal rearrangement, cell movement, and extravasation. We also show that meth decreases expression of transcripts related to antigen presentation, pathogen recognition, and inflammatory responses. Additionally, we found changes in mitochondria related transcripts that could affect the bioenergetics of these cells. These changes could result in increased transmigration of mature monocytes into the CNS, cause dampening of the immune response, which can impair the ability of these cells to fight pathogens, and impact proper functioning of monocytes. These effects on mature monocytes can contribute to the pathogenesis of HIV-NCI in PWH who use meth.

## Materials and methods

### Monocyte isolation and culture

Leukopaks from anonymous healthy donors were obtained from New York Blood Center. Institutional Review Board (IRB) approval for these studies was obtained from the Einstein Human Research Protection Program (HRPP) at Albert Einstein College of Medicine (IRB no. 1994-0003). Isolation and culture of primary mature monocytes was performed as previously described (28). Briefly, peripheral blood mononuclear cells (PBMC) were isolated from leukopaks using Ficoll-Paque PLUS (GE Healthcare, Uppsala, Sweden) density centrifugation. Monocytes were positively selected from the PBMC using Miltenyi Beads CD14 Selection Kit (Miltenyi Biotec, Bergisch Gladbach, Germany) according to manufacturer’s instructions. Isolated CD14+ monocytes were resuspended at a concentration of 2x10^6^ cells/ml in monocyte media containing RPMI 1640 1X (Gibco, Grand Island, New York) supplemented with 5% fetal bovine serum (Sigma-Aldrich, St. Louis, Missouri), 10% human serum (Sigma-Aldrich, St. Louis, Missouri), 1% HEPES (Sigma-Aldrich, St. Louis, Missouri), 1%Pen-Strep (Gibco), and 10ng/ml macrophage colony stimulating factor (M-CSF) (Peprotech, Rocky Hill, New Jersey). Cells were cultured non-adherently in Teflon coated flasks at 37°C with 5% CO_2_ for 2 days to increase the population of mature monocytes in culture (28, 33, 42). Using this culturing technique, we previously showed that the number of mature monocytes is increased from 5-10% to approximately 70-90% (33).

### HIV infection and meth treatment for RNA sequencing

Mature monocytes were divided into 2 flasks, and cells in each flask were resuspended at 10x10^6^ cells/ml in fresh monocyte media and infected for 8 h with 1 ug p24/ml of HIV_ADA_ isolate prepared as described (43). This technique facilitates virus uptake by the cells *in vitro* as established previously in our laboratory (43). Virus was removed from cells by centrifugation, cells were resuspended at 2x10^6^ cells/ml in fresh media, and cultured non-adherently for 3 additional days to facilitate viral infection and replication. Three days post-infection, 50µM Methamphetamine hydrochloride, resuspended in sterile ddH_2_0 (Sigma-Aldrich), was added to one of the flasks for 6 h. This time point is in the range within when meth reaches peak concentration in plasma of meth users (44). The meth concentration chosen is within the range of levels found in plasma of people who use meth (45). After treatment, cells from both flasks were centrifuged for use in flow cytometry and for RNA collection. Supernatants were also collected for HIV p24 quantification using a sensitive HIV p24 alphaLISA kit (Perkin Elmer, Waltham, Massachusetts) as per manufacturer’s protocol to confirm infection of the mature monocytes.

### Mature monocyte quantification by flow cytometry

A portion of both meth treated and untreated HIV-infected cells were analyzed for CD14 and CD16 surface expression by flow cytometry. Cells were labeled on ice in the dark for 30 minutes using anti-human CD14 (0.05 ug per 2x10^5^ cells) conjugated with allophycocyanin (APC) (BD Biosciences, San Jose, California; clone M5E2), and anti-human CD16 (0.05 ug per 2x10^5^ cells) conjugated to phycoerythrin/Cy7 (PE-Cy7) (BD Biosciences; clone 3G8). Corresponding isotype matched antibodies and fluorescence minus one (FMO) were used as negative controls. Labeled cells were fixed with 2% paraformaldehyde in 1%BSA/PBS and acquired with the Attune NxT flow cytometer (ThermoFisher Scientific). A minimum of 10,000 events was acquired for each condition. Data analyses were performed using FlowJo software version 10.6.1 (Treestar, Ashland, Oregon).

### RNA extraction and quality control

RNA extraction was performed using Qiagen RNeasy Micro Kit (Qiagen, Hilden, Germany) as per manufacturer’s protocol. To ensure minimal organic contamination, 260/230 ratios were quantified with Nanodrop (ThermoFisher Scientific). RNA quality and concentration were confirmed by Genewiz (South Plainfield, New Jersey). RNA degradation was measured using TapeStation electrophoresis system (Agilent, Santa Clara, California) and RNA was quantified by Qubit fluorimeter (ThermoFisher Scientific). Good quality RNA was determined by an RNA Integrity Number (RIN) of greater than 6. All samples had RIN > 9 (data not shown).

### Library preparation and RNA sequencing

Library preparation, RNA sequencing, and sequence alignment was performed at Genewiz. Briefly, an RNA-sequencing library was prepared using ribosomal RNA depletion followed by fragmentation and random priming, cDNA synthesis, end repair, 5’ phosphorylation, dA-tailing, adapter ligation, PCR enrichment and sequencing. Sequencing was performed using Illumina HiSeq (Illumina, San Diego, California) with a configuration 2 X 150bp. The sequenced reads were trimmed using Trimmomatic version 0.36. The trimmed reads were mapped onto the *Homo sapiens* GRCh38 reference genome available on ENSEMBL using the STAR aligner version 2.5.2b. The BAM files were generated, and gene hit counts were calculated by using featureCounts from the Subread package version 1.5.2.

### Analyses of differentially expressed genes

Analysis was conducted using R version 4.0.2. We used ComBat_seq function for batch correction to account for the variability inherent in primary cells. Data were then normalized using the calcNormFactors function to account for library size differences among samples. EdgeR function was used to obtain differentially expressed genes (DEG) between meth treated HIV-infected mature monocytes and untreated HIV-infected cells. DEG were considered significant if Benjamini-Hochberg adjusted p-value was less than 0.05 and absolute log 2-fold change was greater than 0.25. To visualize the expression of transcripts between treated and untreated cells, we used ggplot, and heatmap.2 functions.

### Pathway and functional analysis

We performed downstream pathway and functional analyses of the significant DEG using Ingenuity Pathway Analysis (IPA) software version 01-20-04 (Qiagen). We used a p-value of < 0.05 and z-score of either >2 or <-2 to determine significantly predicted affected pathways and functions. GraphPad Prism Software version 9.3.1 (GraphPad Software, San Diego, California) was used to plot the pathway and functional analyses data.

### Western blotting

Monocytes were isolated, cultured, infected, and treated as described. Some HIV-infected mature monocytes were also treated with meth for 24 h for protein analyses to capture proteins translated after the 6 h time point used for RNA analyses. Cells were lysed with RIPA buffer containing 1X Halt Protease Inhibitor and Phosphatase Inhibitor cocktail (Thermo Fisher Scientific). Lysate protein concentrations were quantified by Bradford Assay using Protein Assay Reagent Concentrate (Bio-Rad, Hercules, California). Equal amounts of protein from each condition for each donor were electrophoresed by SDS-PAGE under reducing conditions, with subsequent transfer to polyvinylidene difluoride (PVDF) membranes overnight at 4°C. Li-Cor Revert Total Protein Stain (Li-Cor, Lincoln, Nebraska) was used to quantify total protein optical density (O.D.), using the Li-Cor Odyssey Fc System for visualization, and Image Studio software (Li-Cor) version 5.2 for O.D. quantification. Membranes were blocked in SuperBlock Blocking Buffer (ThermoFisher) followed by overnight incubation at 4°C with mouse anti-MMP9 (1:500, ThermoFisher, #MA5-15886) or rabbit anti-Gelsolin (1:1000, Cell Signaling Technology, Danvers, Massachusetts, #12953) in 5% BSA-TBST. HRP-conjugated anti-mouse IgG (Cell Signaling Technology, #7076) was used as secondary antibody for MMP9, and HRP-conjugated anti-rabbit IgG (Cell Signaling Technology, #7074) was used for gelsolin, each at 1:1000 dilution in 5% milk-TBST. Blots were developed using Super Signal West Femto Chemiluminescent Substrate (ThermoFisher) for MMP9, or Western Lightning Plus ECL Oxidizing Reagent Plus (Perkin Elmer) for gelsolin, and visualized and analyzed the same as for total protein. The O.D. of target proteins were normalized to total protein O.D. Data were analyzed as the fold change of normalized O.D. of target proteins in HIV-infected meth treated cells over the normalized O.D. of target protein in untreated cells. Figures were prepared in GraphPad Prism Software version 9.3.1.

### MMP-9 ELISA

Cells infected and treated with meth as described were centrifuged at 1000rpm for 5 minutes at room temperature. The supernatants were collected and assayed using the MMP-9 DuoSet ELISA kit as per manufacturer’s instructions (R&D Systems, Minneapolis, Minnesota). The limit of detection for this kit is 31.25pg/ml. GraphPad Prism Software version 9.3.1 was used to prepare the figures and statistical analyses. The MMP-9 concentrations were normalized to 10 million cells to adjust for the different cell numbers in culture per experiment. Data were analyzed as the fold change of HIV-infected meth treated cells over untreated HIV-infected cells.

### Statistical analyses

For MMP-9 ELISA and HIV p24 alphalisa experiments, D’Agostino-Pearson omnibus normality test was performed to test for gaussian distribution of the data. One sample t-test or paired t-test was used for the normally distributed data, with p < 0.05 considered statistically significant.

## Results

### Meth induces differential gene expression in HIV-infected mature monocytes

**Figure 1:**
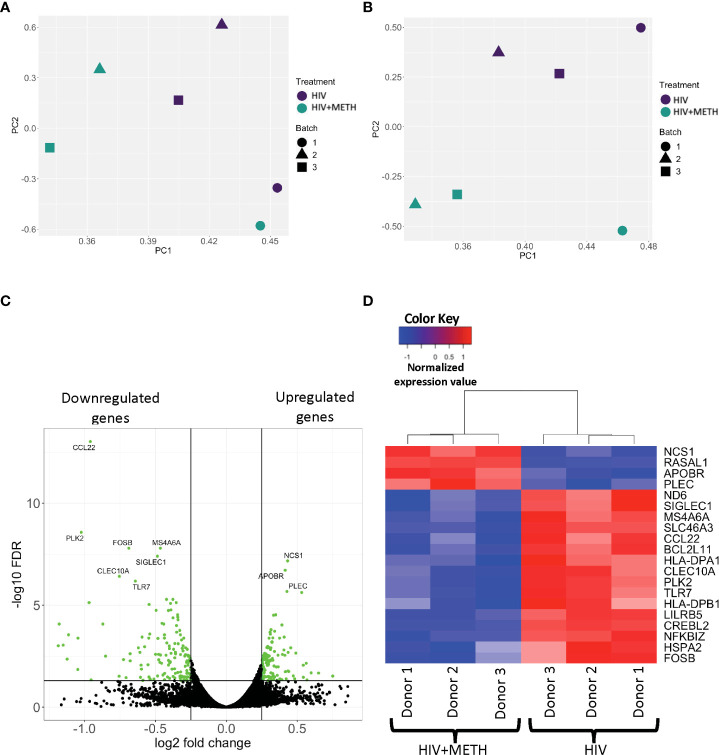
RNA sequencing analyses demonstrate differential gene expression in HIV-infected mature monocytes treated with meth compared to untreated HIV-infected cells. **(A, B)** Principal component analysis (PCA) plot of sample clustering based on gene expression patterns before **(A)** and after **(B)** batch correction. Each data point represents an individual sample, and the data shapes represent the individual donors. The y-axis and x-axis represent the first and second principal components, respectively. HIV-infected mature monocytes treated with meth are shown in blue and untreated HIV-infected mature monocytes are shown in green. **(C)** A volcano plot showing overall DEG between the HIV-infected mature monocytes treated with or without meth. Each dot represents a gene that was either statistically significantly different(green) or unchanged (black). The y-axis represents -log false discovery rate (FDR), and the x-axis represents log 2-fold change. **(D)** A heat map representing normalized gene expression levels of the top 20 DEG between HIV-infected mature monocytes treated with meth or untreated. Each column represents an individual sample, and each row represents a gene. The color scale represents lower (blue) to higher (red) gene expression levels.

The mechanisms by which meth contributes to HIV-NCI in PWH are not incompletely characterized. We examined the effects of meth on the transcriptome of HIV-infected mature monocytes by RNA-sequencing to identify potential mechanisms by which meth contributes to neuropathogenesis in PWH. Primary monocytes from three independent healthy donors were positively isolated from leukopaks, cultured and infected with HIV, and treated with meth as described. RNA from HIV-infected and HIV-infected meth treated cells was then sequenced, mapped onto the genome, counted, and then we performed principal component analysis (PCA) to determine similarities in gene expression patterns among samples. Separate samples from the same donors clustered together (**Figure 1A**). To reduce the bias in downstream differential gene expression analyses that could be introduced by this similar clustering, we performed batch correction that accounts for the donor variation commonly seen in primary cells from different people (**Figure 1B**). We identified 121 upregulated transcripts and 137 downregulated transcripts in HIV-infected mature monocytes treated with meth relative to untreated infected cells (**Figure 1C** and **Table S1**). Some of the top upregulated transcripts are involved in cell movement signaling pathways including *NCS1*, a regulator of G-protein coupled receptor phosphorylation, and *RASALI*, a GTPase-activating molecule (**Figure 1D**). Meth also upregulated cytoskeletal binding molecules including *PLEC* (**Figure 1D**). Meth downregulated transcripts including *ND6*, a mitochondria molecule involved in oxidative phosphorylation, and *TLR7*, a toll like receptor (TLR) involved in antiviral immune response (**Figure 1D**). Meth also downregulated transcripts including *HLA-DPA1* and *HLA-DPB1* that are important in antigen presentation. This differential gene expression profile induced by meth could lead to changes in functions of HIV-infected mature monocytes contributing to increased cell migration, dysregulated immune response, and consequently neuropathogenesis in PWH who use meth.

To ensure that meth did not affect cell survival or mature monocyte surface markers, a portion of infected monocytes was analyzed by flow cytometry for CD14 and CD16. Meth treatment did not change the survival, nor the percentage of mature monocytes (**Supplementary Figures 1A, B**). We confirmed HIV infection by quantifying HIV p24 protein in cell supernatant with AlphaLISA. Meth increased the amount of HIV released from cells from each independent donor (**Supplementary Figure 2**). This is consistent with other studies that reported increased HIV infection in other cell types including macrophages (46, 47).

### HIV-infected meth-treated mature monocytes are predicted to have differential gene expression related to cell activation, infection by viruses, adhesion and extravasation, and cytoskeletal rearrangement

**Figure 2:**
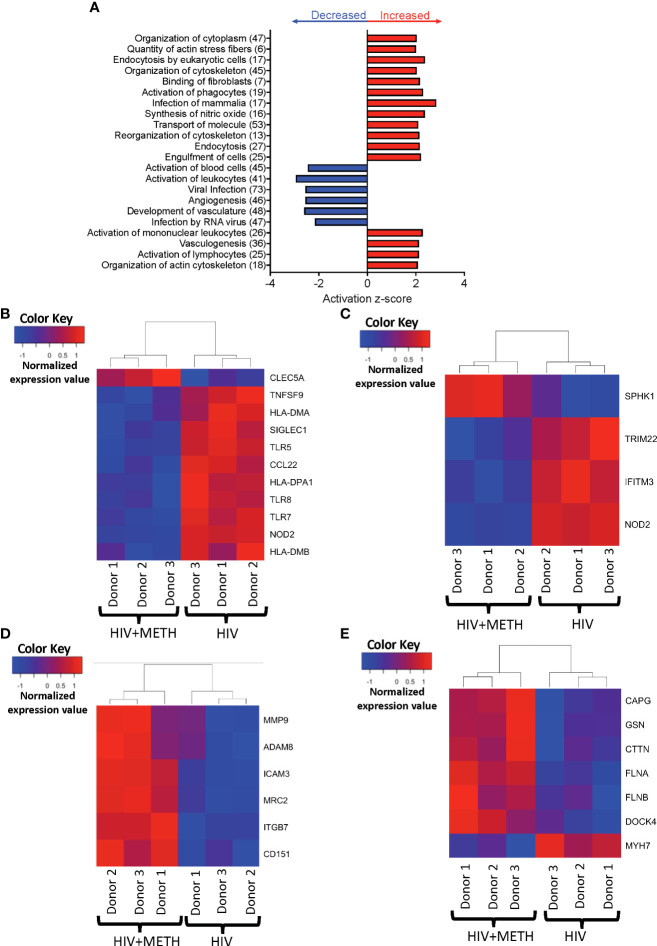
HIV-infected mature monocytes treated with meth compared to untreated HIV-infected cells have a predicted increase in viral infection and cytoskeletal rearrangement and decrease in cell activation. **(A)** Top functions predicted to be increased (red) or decreased (blue) in the meth treated compared to untreated HIV-infected cells. The y-axis represents the list of functions, and the numbers in the parenthesis represent the quantity of molecules that were differentially expressed after meth treatment. The x-axis represents activation z-score. **(B–E)** Heat maps representing normalized expression levels of some genes involved in **(B)** activation of leukocytes, **(C)** viral infection, **(D)** cell adhesion and matrix degradation, and **(E)** organization of cytoskeleton in HIV-infected mature monocytes treated with and without meth. Each column represents an individual sample, and each row represents a gene. The color scale represents lower (blue) to higher (red) gene expression levels. The dendrograms show unsupervised clustering of samples.

We performed IPA of DEG to identify predicted functions of mature monocytes that are impacted by meth. We found that meth downregulated pathogen recognition and binding molecules *TLR5*, *TLR7*, *TLR8*, and *SIGLEC1*, antigen presenting MHC transcripts *HLA-DMA*, *HLA-DMB*, and *HLA-DPA1*, and *TNFSF9*, *NOD2*, and *CCL22*, molecules involved in inflammatory responses (**Table S2** and **Figure 2A, B**). Downregulation of these transcripts by meth could lead to dysregulated immune responses by impairing the ability of monocytes to respond to pathogens and other inflammatory related stimuli. Infection by virus was predicted to be activated in meth treated HIV-infected mature monocytes compared to untreated infected cells (**Table S2** and **Figure 2A**). Some of the transcripts related to infection by virus that were decreased include *TRIM22*, *NOD2*, *IFITM3*, and *SPHK1* (**Figure 2C**). These changes could lead to a decreased immune response to fight viruses, leading to increased viral infection in the meth treated HIV-infected mature monocytes that can contribute to viral reservoir persistence.

Our data showed meth increased transcripts involved in cell adhesion and matrix degradation, including *MMP-9, MRC2*, *ADAM8, ITGB7*, *CD151* and *ICAM3* (**Table S1** and **Figure 2D**). We also found that meth increased expression of molecules involved in cytoskeletal arrangement including *MYH7*, *MYH8, CTTN*, *GSN*, *FLNA*, *FLNB*, *DOCK4*, and *CAPG* (**Table S2** and **Figure 2E**). The proteins encoded by these transcripts are important in cytoskeletal rearrangement that is needed for cell motility and invasion. These changes may result in increased HIV-infected mature monocyte migration into the CNS. The entry of these cells into the CNS can lead to infection and activation of other CNS cells, and production of inflammatory mediators including host and viral proteins, that can contribute to neuronal damage and HIV-NCI.

### HIV-infected meth-treated mature monocytes have dysregulated transcripts involved in inflammatory signaling and cellular metabolic pathways

**Figure 3:**
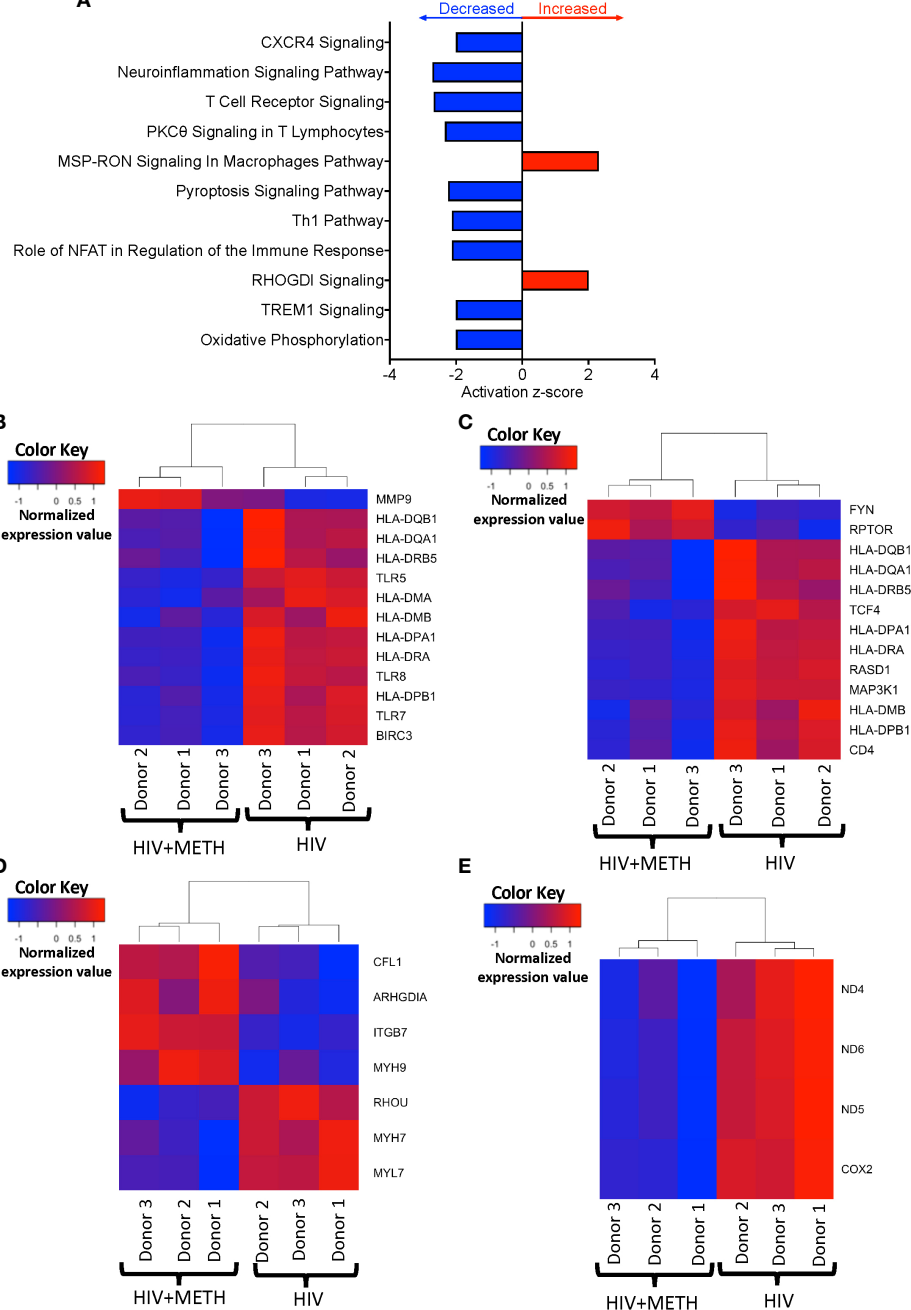
HIV-infected mature monocytes treated with meth have decreased expression of genes related to antigen presentation, pathogen recognition, and oxidative phosphorylation, and upregulation of genes related to cellular RHO-GD1 signaling pathways. **(A)** Top cellular pathways predicted to be increased (red) or decreased (blue) in meth treated compared to untreated HIV-infected cells. The y-axis represents the list of pathways, and the x-axis represents activation z-score. **(B–E)** Heat maps representing normalized expression levels of some genes involved in **(B)** neuroinflammation signaling pathways, **(C)** T-cell receptor signaling, **(D)** oxidative phosphorylation, and **(E)** RHO-GDI signaling in HIV-infected mature monocytes treated, or not, with meth. Each column represents an individual sample, and each row represents a gene. The color scale represents lower (blue) to higher (red) gene expression levels. The dendrograms show unsupervised clustering of samples.

We determined potential biological pathways that may be changed in HIV-infected mature monocytes treated with meth using IPA of the DEG. We found that pathways including neuroinflammation signaling, T-cell receptor signaling, and oxidative phosphorylation were predicted to be downregulated, whereas RHOGDI and MSP-RON signaling pathways were predicted to be upregulated (**Figure 3A**, **Table S3**). We further examined transcripts involved in neuroinflammation signaling pathways and signaling with T-cell receptor. We found that molecules such as Major Histocompatibility Complex (MHC) class II molecules including *HLA-DMA*, *HLA-DMB*, *HLA-DPA1*, *HLA-DQB1*, *HLA-DRA*, and *HLA-DRB*, and toll like receptor transcripts, *TLR5*, *TLR7*, and *TLR8*, were downregulated by meth (**Figures 3B, C**), which is consistent with predicted pathway changes. We also found meth downregulated *CISD2* and *VMP1* which are involved in the endosomal-lysosome pathway as part of antigen processing (**Table S1**). These suggest that meth may impair the ability of HIV-infected mature monocytes to recognize, process, and present antigens to T-cells. This could lead to viral immune evasion and persistence in the CNS. We also found that mitochondrial transcripts *COX2*, *ND4*, *ND5, and ND6* were downregulated contributing to the predicted decrease in oxidative phosphorylation (**Figure 3D**). Additionally, we found downregulation of the ATP synthase transcript, *ATP8*, and upregulation of transcripts involved in glycolysis, such as *ENO2* and *PFKP* (**Table S1**). These could lead to bioenergetic changes in the monocytes, and potentially affect monocyte functions such as fighting pathogens and immune surveillance. Molecules involved in RHOGDI signaling including *CFL1*, *ITGB7*, *MYH7*, *MYH9*, and *RHOU*, and MSP-RON pathways such as *ST14* were upregulated by meth (**Figure 3E** and **Table S2**). The increased expression of these molecules could lead to downstream activation of signaling molecules that may contribute to changes in the biology of these monocytes.

### Meth increases MMP-9 protein in HIV-infected mature monocytes

**Figure 4:**
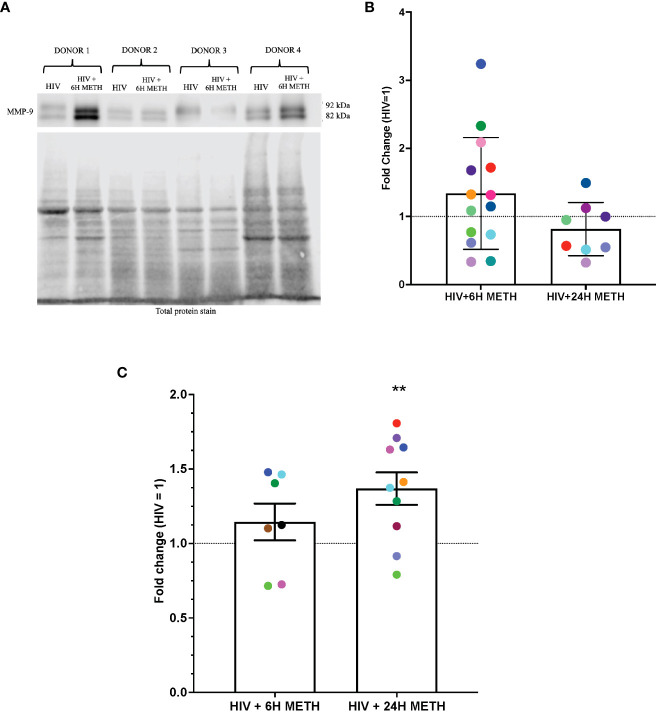
MMP-9 protein is increased in HIV-infected mature monocytes treated with meth. **(A)** A representative western blot with corresponding total protein stain of HIV-infected mature monocytes either untreated or treated for 6 h with meth. **(B)** Fold change of normalized MMP-9 protein from HIV-infected mature monocytes treated with meth over untreated HIV-infected mature monocytes. Each colored dot represents an individual donor. The columns and error bars depict mean and standard deviation (SD), respectively. n=7–14. **(C)** Fold change of normalized MMP-9 protein in supernatants of HIV-infected mature monocytes treated with meth over untreated HIV-infected cells. Each colored dot represents an individual donor. The columns and error bars depict mean and standard error of the mean (SEM), respectively; n=7-10; **, p<0.005 by the one sample t-test.

IPA identified cytoskeletal rearrangement as a function predicted to be increased by meth in mature monocytes. One specific DEG that was increased by meth in this pathway is Matrix metalloproteinase-9 (MMP-9) (**Table S1**). We validated protein expression of MMP-9 because metalloproteinases have been shown to be important in neuropathogenesis (48). Animal studies showed that blocking MMP-9 reduced neuroinflammation in multiple sclerosis animal model (49). MMP-9 degrades extracellular matrices and may contribute to neuroinflammation by increasing monocyte migration into the CNS (50), and/or by activating chemokines such as CXCL8 that can contribute to leukocyte infiltration (49). To validate this RNA-seq finding, we determined protein expression of MMP-9 in HIV-infected mature monocytes treated with or without meth, by Western blotting and by ELISA. MMP-9 can exist as pro- and active forms. We found that intracellular levels of both pro- and active MMP-9 in HIV-infected mature monocytes treated with meth for 6 h appeared to be increased (**Figures 4A, B**). There was no difference in MMP-9 after 24 h of meth treatment (**Figure 4B**). While the increase in intracellular MMP-9 at 6 hours of meth was not reflected by an increase in extracellular MMP-9 by ELISA (**Figure 4C**), we found that extracellular MMP-9 was statistically increased after 24 h meth treatment relative to untreated infected cells (**Figure 4C**). It could be that after 24 h of meth treatment, HIV-infected mature monocytes are secreting more MMP-9 into the extracellular environment than what is being maintained intracellularly, such that increased protein level cannot be detected intracellularly at that time point. Increased MMP-9 could contribute to increased transmigration of mature monocytes across the BBB into the CNS by degrading the basement membrane, and by increasing recruitment of mature monocytes by increasing chemokine activation.

### Meth appears to increase gelsolin in HIV-infected mature monocytes as determined by western blot analyses

**Figure 5:**
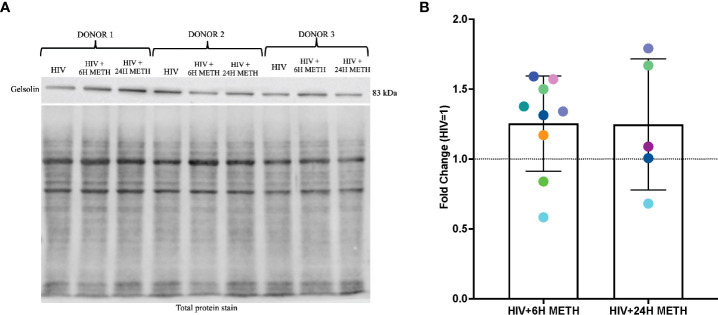
Gelsolin protein is increased in HIV-infected mature monocytes treated with meth. **(A)** A representative gelsolin western blot with corresponding total protein stain of HIV-infected mature monocytes either untreated or treated for 6 h or 24 with meth. **(B)** Fold change of gelsolin of HIV-infected mature monocytes treated with meth over untreated HIV-infected cells. Each colored dot represents an individual donor. The column and error bars depict mean and standard deviation (SD), respectively. n = 5-9.

IPA analyses of DEG also showed that organization of actin cytoskeleton and cell movement of mature monocytes treated with meth was predicted to be increased. One transcript in this pathway that was increased by meth is *GSN* (**Table S1**), which encodes for Gelsolin, an actin binding protein involved in severing and capping actin filaments, an important step in cytoskeletal rearrangement needed for cell movement (51). We validated RNA-seq data by performing western blotting of gelsolin, in HIV-infected cells treated with or without meth. We validated gelsolin expression because it is one of the most abundant actin binding proteins that regulate cytoskeletal arrangement, a necessary process in monocyte migration (52). We found that 6 h of meth treatment increased intracellular gelsolin in 7 out of 9 donors (**Figures 5A, B**). While there is donor variability in gelsolin after 24 h meth treatment, there is a trend towards increased gelsolin (**Figures 5A, B**).

**Figure 6:**
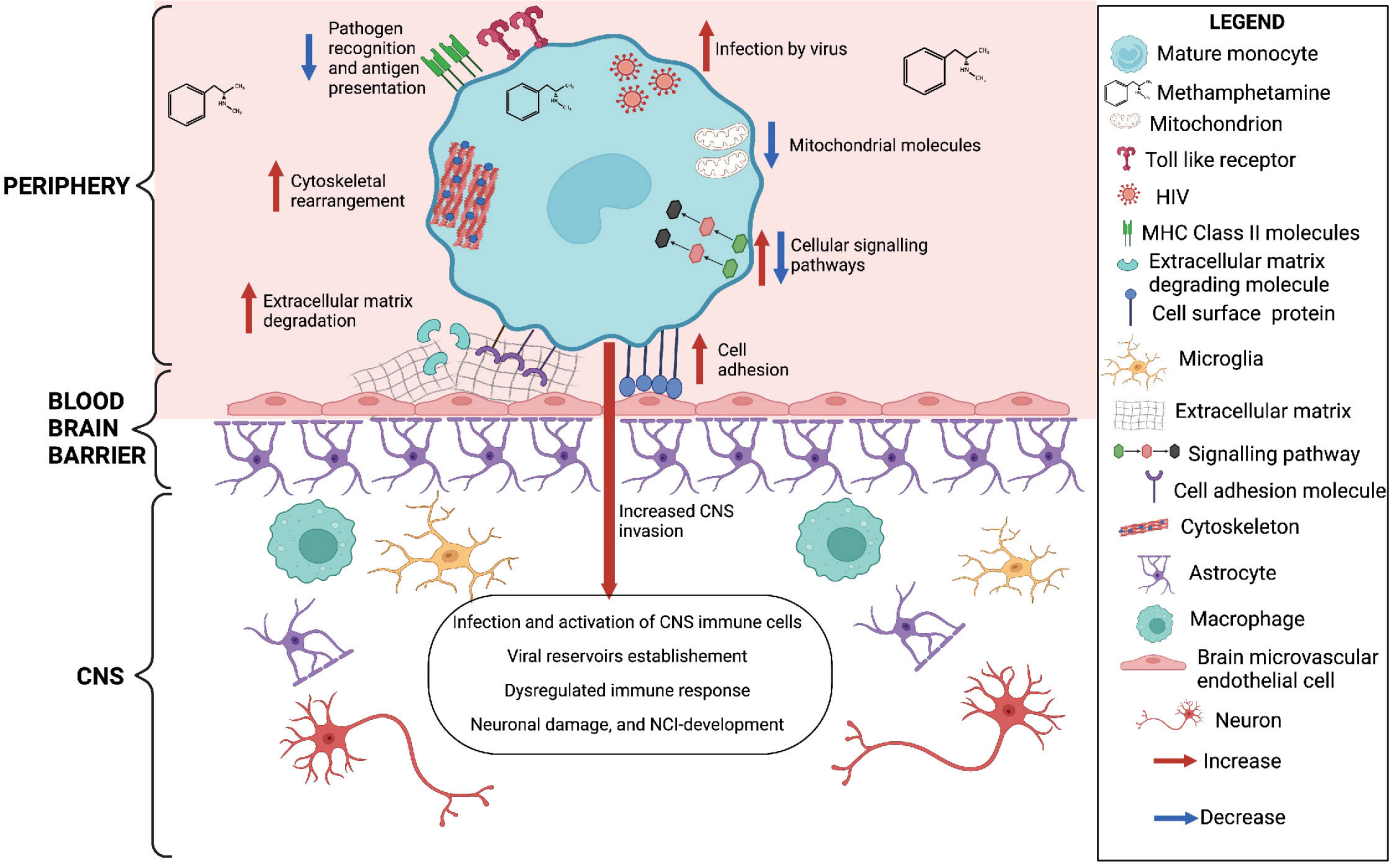
Meth-mediated effects on HIV-infected mature monocytes as identified by RNA sequencing and western blot analyses. Figure created with BioRender.com.

In summary, our results show that meth may contribute to increased monocyte recruitment, through increased expression of molecules related to matrix degradation, cell adhesion and cytoskeletal rearrangement (**Figure 6**). We also identified downregulation of transcripts involved in antigen presentation and pathogen recognition, and predicted increase in viral infection (**Figure 6**). These changes can result in immune dysregulation and increased viral reservoirs once these cells enter the CNS. Meth also induced changes in signaling molecules, and downregulated molecules involved in oxidative phosphorylation (**Figure 6**). These changes could impact the functions of these cells, contributing to ongoing neuropathogenesis in PWH who use meth.

## Discussion

Despite the great success of ART, comorbidities including HIV-NCI persist in PWH who use meth (53). However, the mechanisms by which meth contributes to HIV-NCI have not been fully described. We characterized the impact of meth on HIV-infected mature monocytes using RNA sequencing analyses to identify molecules that are potentially involved in the development HIV-NCI in PWH who use meth. We identified differential gene expression in HIV-infected mature monocytes treated with meth compared to untreated HIV-infected cells. Some DEG include those related to increased cell adhesion, extravasation, cytoskeletal rearrangement, and viral infection of mature monocytes. We also found downregulation of DEG involved in antigen presentation, pathogen recognition, inflammatory response, and oxidative phosphorylation.

HIV-infected monocyte migration into the CNS contributes to viral entry and inflammation which contributes to HIV-NCI. Our study demonstrates that meth treatment of HIV-infected mature monocytes led to the upregulation of transcripts involved in matrix degradation and cell adhesion. MMP-9 is an extracellular protease that breaks down extracellular matrices and BBB basement membranes. This has been shown to lead to BBB disruption and immune cell recruitment into the CNS (50). We found increased MMP-9 RNA and protein in HIV-infected mature monocytes treated with meth compared to untreated HIV-infected cells alone. We also show that meth increases the amount of MMP-9 released into the extracellular environment by HIV-infected cells. Our data are in agreement with findings from other studies that showed that meth increased expression of MMP9 in mice, and of MMP-9 protein in brain microvascular endothelial cells (54, 55). In addition to our western blotting and ELISA data, the increased expression of MMP9 is also supported by our *in vitro* data showing that meth increases transmigration of HIV-infected mature monocytes across a human BBB model in response to CCL2, a chemokine elevated in the CNS of PWH (data not shown). We found increased levels of *MRC2* and *ADAM8* in meth treated HIV-infected cells. MRC2, a member of mannose receptor family, binds and internalizes collagen contributing to matrix degradation and turnover (56). ADAM8, a member of a disintegrin and metalloproteinase family, is involved in integrin activation, cell adhesion, migration, and break down of extracellular matrix components to promote cell movement (57). ADAM8 has also been shown to increase cell migration through upregulation of MMP-9 (58). The increased levels of *MMP-9*, *MRC2* and *ADAM8* after meth treatment may contribute to increased degradation of extracellular matrix and basement membrane components, and consequently migration of HIV-infected mature monocytes into the CNS. This may lead to accumulation of infected cells contributing to viral reservoirs and CNS damage.

Monocyte adhesion to brain microvascular endothelial cells is important during monocyte transmigration into the CNS. We show that meth increases expression of the cell adhesion-related transcripts *ITGB7*, *ICAM3*, *CD151*, and *ICAM3*. *ITGB7* encodes a protein that heterodimerizes with either CD49d or CD103 to form α_4_β_7_ and α_E_β_7_ integrins, respectively. These integrins bind with their ligands MADCAM-1 and E-cadherin, respectively, promoting cell adhesion (59). *ITGB7* downregulation was reported to decrease adhesion and migration of multiple myeloma cells (60). In addition, ITGB7 is important in T-cell homing in the gut and is involved in the pathogenesis of inflammatory bowel disease (61). ICAM-3 is an adhesion molecule that can interact with LFA-1 extracellularly or ezrin/radixin/moesin intracellularly, contributing to tumor metastasis (62). CD151, a tetraspanin, interacts with integrins promoting cell matrix adhesion (63). In T-cells, CD151 interacts with integrins LFA-1 and VLA-4, and upon external stimuli, it can lead to actin rearrangement and cell migration (64). In mature monocytes, CD151 is involved in formation of multinucleated giant cells that can occur during inflammatory processes (65). CD151 also forms homotypic interactions that lead to increased cell movement and MMP-9 production (66). CD151 can interact with JAM-A, a molecule increased on the surface of HIV-infected mature monocytes, contributing to increased cell migration (33, 67). Changes in these molecules may contribute to cell adhesion, downstream signaling activation, and their consequent migration from the periphery into the CNS.

Actin cytoskeleton rearrangement is key for monocyte transmigration into the CNS across the BBB. Myosin II, a motor protein that binds F-actin, contributes to cell attachment, spreading and migration (68). We found that meth upregulates myosin II heavy chain molecule, *MYH9*, and there is a predicted increase in cytoskeletal rearrangement. We also show there is an increase in *CFL1*, which encodes cofilin. Cofilin is an actin binding protein that cleaves F-actin providing ends for polymerization and depolymerization and is important in chemotaxis and directional cell movement (69, 70). This could lead to increased actin rearrangement that contributes to cell movement. Our findings are in agreement with another study that showed increased expression of cofilin with activation of RhoA/ROCK signaling pathway leading to cytoskeletal rearrangement in rat brain microvascular endothelial cells treated with meth (55).

Meth treatment of HIV-infected mature monocytes also upregulated *FLNA* and *GSN*, which encode filamin A and gelsolin, respectively. Filamins are actin binding and cross-linking proteins that facilitate cell spreading and migration (71, 72). In neutrophils, a decrease in Filamin A resulted in decreased cell migration and reduced myosin-II activation (73). Gelsolin is an actin binding protein implicated in migration and invasion of cancer cells (74, 75). This protein severs and caps F-actin, which is needed for actin polymerization during cell movement (51). We found increased gelsolin RNA and demonstrated a trend towards increased gelsolin protein in HIV-infected mature monocytes treated with meth compared to untreated cells. One study showed that mouse macrophages that do not express gelsolin have decreased chemotaxis and migration, suggesting this molecule is important for myeloid cell migration (76). In mouse osteoclasts, gelsolin promotes podosome formation and cell motility (77). Meth-induced changes in the expression of actin binding proteins could contribute to changes in cytoskeletal arrangement mediating cell movement and entry into the CNS.

Several signaling pathways regulate monocyte functions such as movement and differentiation that may contribute to HIV neuropathogenesis. Our analyses showed changes in expression of transcripts related to signaling pathways and predicted an increase in RHOGDI signaling. We found increased expression of *DOCK4*, a guanine exchange factor, in meth treated HIV-infected cells. DOCK4 activates Rac1 and promotes actin reorganization and formation of lamellipodia at the leading edge of breast cancer cells (78). Our results also showed an increased expression of *NCS1*, a molecule that encodes a calcium binding molecule that regulates G protein coupled receptor phosphorylation and promotes cell movement (79). We found a predicted activation of the MSP-RON pathway in meth treated HIV-infected mature monocytes. Macrophage stimulating protein (MSP) is activated by matriptase, encoded by *ST14*, a transcript upregulated by meth. This activated form binds to Recepteur d’origine Nantais (RON), resulting in increased cell migration and matrix invasion (80). Matriptase mediated downstream protein kinase C (PKC) signaling that led to increased MMP-9 and metastasis of a breast cancer cell line (81). Dysregulation of these molecules can change signaling pathways in mature monocytes that facilitate their entry into the CNS.

Mature monocytes are involved in immune responses through their ability to process and present antigens, activate T-cells, and release inflammatory mediators in the CNS. Once in the CNS, monocytes can also differentiate into perivascular macrophages that contribute to antigen presentation and inflammatory responses (82). Our analyses showed that meth treatment of HIV-infected mature monocytes decreases expression of many antigen presenting MHC class II molecules (*HLA-DMA*, *HLA-DMB*, *HLA-DPA1*, *HLA-DPB1*, *HLA-DQA1*, *HLA-DQB1*, *HLA-DRA*). These are important for processing and presenting antigens derived from extracellular pathogens (83). Downregulation of MHC class II molecules can reduce the ability of the mature monocytes to present antigens to CD4^+^ T-cells, compromising immune response to pathogens. We also show decreased *CISD2* and *VMP* transcripts, involved in endosomal-lysosomal pathways important for antigen presentation by MHC molecules (84–86). Our findings are consistent with a study that showed that meth decreases MHC Class II antigen processing and presentation by inhibiting the endosomal-lysosomal pathway in dendritic cells (87). Although a separate report showed that the ability of MHC Class II antigen processing and presentation is not impaired in total monocytes from PWH, our data indicate that in the presence of meth, this may be dysregulated in HIV-infected mature monocytes (88). This could inhibit clearance of pathogens in the CNS.

Toll like receptors (TLR) are pattern recognition receptors (PRR) present on immune cells including monocytes. TLR are important for inflammation and immune responses to pathogens. They are activated upon binding of pathogen-associated molecular patterns, resulting in downstream signaling that leads to interferon production. This can mediate recruitment of additional immune cells and activation of antiviral immune responses (89). Our data show that meth downregulates expression of *TLR7*, an endosomal receptor for single stranded RNA (89, 90). TLR7 was shown to trigger a Type I interferon response upon HIV RNA binding in dendritic cells (91, 92). Meth also decreases TLR8, another endosomal receptor for single stranded RNA (89, 90). TLR8 in monocytes recognize HIV, leading to inflammasome activation (91, 92). Meth-mediated downregulation of TLR7 and TLR8 in HIV-infected mature monocytes could be impaired, resulting in reduced ability to clear HIV after entering the CNS. In addition, activating TLR signaling pathways is a potential mechanism for reactivation of latent virus within viral reservoirs in eradication approaches (93–95). A TLR8 receptor agonist was shown to re-activate latent HIV in CD4 T-cells (95). Downregulation of *TLR8* by meth in HIV-infected mature monocytes could reduce efficacy of this strategy in PWH who use meth.

Our functional analyses predicted an increase in viral infection of meth treated HIV-infected mature monocytes compared to untreated HIV-infected cells. We found increased levels of HIV release from cells after 6h of meth treatment, although we did not quantify intracellular HIV levels. The early changes in transcripts expression related to infection by viruses could contribute to increased HIV infection of meth treated mature monocytes at later timepoints. This is consistent with studies that reported increased HIV infection in meth treated macrophages, monocytes, microglia, and dendritic cells treated with meth for various amounts of time (46, 96–99). The prediction of increased viral infection may be due, in part, to downregulation of the antiviral related molecules *IFITM3*, and *TRIM22*. Interferon-induced transmembrane proteins (IFITM) can inhibit HIV entry and viral protein synthesis, and IFITM3 was reported to be involved in antiviral responses by trafficking vesicles containing viruses into the lysosome for degradation (100–102). Downregulation of *IFITM3* by meth could result in increased viral infection of mature monocytes due to impaired ability to reduce viral entry and/or degradation of the virus. TRIM22, a member of tripartite motif proteins family, also inhibits HIV replication (103–106). Thus, downregulation of *TRIM22* in mature monocytes can promote increased viral production.

Under homeostatic conditions, cells depend upon mitochondrial oxidative phosphorylation as the main source of energy. We found decreased expression of mitochondria Complex I gene transcripts, *ND4*, *ND5*, and *ND6*, and the ATP synthase transcript, *ATP8*, in meth treated HIV-infected mature monocytes, and a predicted decrease in the oxidative phosphorylation pathway. Downregulation of complex I and ATP synthase molecules could lead to decreased energy production through the oxidative phosphorylation pathway (107). Our results are consistent with a study that found that meth causes mitochondria dysfunction through loss of mitochondrial membrane potential, mitochondria permeability transition pore opening, or dysregulation of mitochondria complexes (108). Another study reported that short meth exposure decreases the oxygen consumption rate and ATP levels in astrocytes (109). Our data show upregulation of transcripts encoding enzymes involved in glycolysis, including *ENO2* and *PFKP* in the meth treated cells. These results are similar to those from another study that showed increased protein levels of ENO2 in immature dendritic cells treated with meth (110). Meth-mediated changes in glycolysis related molecules could shift the metabolic profile of the cells to rely on energy produced by glycolysis. A switch to glycolysis results in release of metabolites that cause downstream signaling that can promote inflammation and cell migration (111, 112). This may contribute to influx of cells into the CNS leading to inflammation and consequent neuronal damage.

Mitochondria complexes I and III are major sources of reactive oxygen species (ROS) which are involved in the regulation of hypoxia, inflammation, and cell death (113). We observed a decrease of complex I transcripts expression after meth treatment. Our data also showed that meth caused a downregulation of the antioxidant transcript *SELENOP*, and upregulation of an oxidative stress marker, *GGT1* (114, 115). These changes can lead to dysregulated ROS production, increased oxidative stress, and contribute to CNS inflammation. One study found decreased levels of Complex I proteins in PBMC from PWH and this correlated with inflammation (116). Additionally, increased ROS has been shown in HIV neuropathogenesis (117). The meth-induced changes indicate a potential ROS imbalance, that can contribute to cellular stress and homeostatic imbalance in the CNS.

Meth can induce the differential gene expression patterns in HIV-infected mature monocytes through activation of various downstream signaling pathways. Identifying these pathways will provide potential upstream targets to limit the impact of meth on specific monocyte functions. Meth is a lipophilic molecule that enters cells either by diffusing across the plasma membrane or by binding dopamine transporters, which are expressed on monocytes (118). Inside the cell, meth can bind to trace amine-associated receptor 1 (TAAR1), an intracellular G-protein coupled receptor (119–121). This can lead to downstream activation of RhoA, PKA or CREB signaling pathways (119–121). CREB is a transcriptional factor that regulates transcription of various genes including those of MHC class II molecules (122). CREB also regulates transcription of cofilin, *CFL1*, a molecule whose expression was upregulated in our HIV-infected monocytes treated with meth (123). Meth-mediated modulation of all these pathways may contribute to the differential gene expression patterns identified in our study, which may contribute to changes in cellular functions. Thus, targeting these pathways with inhibitors may limit the meth-mediated effects in HIV-infected monocytes.

To determine whether HIV alone affects expression of some of the DEG identified, we examined our previously published single cell RNA sequencing data to compare expression of the transcripts between HIV-infected and uninfected mature monocytes cultured *in vitro* (42). We showed that HIV alone did not increase expression of many of the cell movement related molecules including *MMP9*, *ADAM8*, *GSN*, and *CFL1*. However, we found that HIV downregulated expression of some MHC class II molecules including *HLA-DMA*, *HLA-DMB*, and *HLA-DPA1* (42). This suggests that both HIV and meth downregulate these molecules that could impair the ability of the cells to present antigens, including viral proteins to protect them from being killed by cytotoxic T cells. This can contribute to persistence of viral reservoirs. Although we did not determine the effects of meth on expression of these molecules on uninfected mature monocytes, our data suggest that in the context of HIV, meth could worsen disease pathogenesis by further impairing some of their functions.

Our study did not determine the percentage of cells expressing the identified DEG. To address this, we used our published single cell RNA sequencing data, and found that there was heterogeneous expression of some molecules (42). For example, *MMP9*, *GSN*, *ND4*, and *HLA-DMA* were present in more than 70% of HIV-infected mature monocytes, whereas molecules such as *ITGB7*, *FLNB*, and *TLR8* were present in fewer than 50% of the cells (42). Future studies can perform flow cytometry and microscopy studies to evaluate protein expression of these molecules in mature monocytes.

Our data identify potential mechanisms by which meth may exacerbate HIV-NCI in PWH. Some of the limitations of this study include the short period of time for which the cells were treated with meth. The gene expression changes found may not represent those that occur during chronic meth use. HIV-infected mature monocytes are a heterogeneous population comprised of HIV harboring and HIV-exposed cells. This study used bulk RNA sequencing. Therefore, we cannot distinguish the effects of meth on cells that are harboring HIV from the effects on cells exposed to HIV but not harboring the virus. It is also possible that some of the specific gene expression changes might not reflect protein or functional changes. Additional studies are needed to determine the effects of chronic meth exposure on HIV-infected mature monocytes. More assays need to be performed to confirm the RNA sequencing data and to determine the specific effect of meth on HIV harboring and HIV-exposed cells. We also did not perform comparisons among untreated uninfected cells, meth treated uninfected cells, untreated HIV-infected cells, and meth treated HIV-infected cells. Thus, we could not determine the effects of meth with and without HIV. Our future studies will address this. Another limitation of this study is we could not sort for specifically CD14^+^CD16^+^ monocytes due to the limited number of cells. However, we showed that the majority of cells were mature monocytes. Future studies will also confirm the identified DEG using flow cytometry and microscopy assays to specifically identify expression of the molecules on cells expressing both CD14 and CD16.

To our knowledge, this is the first study to characterize the impact of meth on HIV-infected mature monocytes, a key cell type in the neuropathogenesis of HIV-NCI. We found that meth may increase monocyte recruitment, viral seeding and reseeding, and immune dysregulation through changes in transcripts involved in matrix degradation, cell adhesion, cytoskeletal arrangement, signaling pathways, antigen presentation, pathogen recognition, viral infection, and oxidative phosphorylation. These data identify potential biological changes that occur in meth treated HIV-infected mature monocytes that can contribute to HIV-NCI in PWH who use meth. These changes could potentially be used for therapeutic targets to reduce infected monocyte entry into the CNS, viral seeding and reseeding, immune response dysregulation, and neuronal damage in PWH who use meth.

## Data availability statement

The data represented in this study are deposited in the GEO repository, accession number GSE210168.

## Ethics statement

The studies involving human participants were reviewed and approved by Einstein Human Research Protection Program (HRPP) at Albert Einstein College of Medicine (IRB no. 1994-0003). Written informed consent for participation was not required for this study in accordance with the national legislation and the institutional requirements.

## Author contributions

JB and VC designed this study. VC performed the computational data analysis and wrote the manuscript. VC, JW, SM-M, LM, LC, and JB designed and performed the validation experiments. VC, JW, SM-M, LM, LC, and JB contributed to discussions about manuscript content, writing, and editing of the manuscript. All authors contributed to the article and approved the submitted version.

## Funding

This work was funded by NIH grants R01DA044584 (VC, LC, and JB), R01DA048609 (LC, SM-M, and JB), R01MH112391 (SM-M and JB) and 5T32AI007501 (JW).

## Acknowledgments

We would also like to thank The Advanced Technologies and Biomarker Core under the Einstein-Rockefeller-CUNY Center for AIDS Research (P30AI124414).

## Conflict of interest

The authors declare that the research was conducted in the absence of any commercial or financial relationships that could be construed as a potential conflict of interest.

## Publisher’s note

All claims expressed in this article are solely those of the authors and do not necessarily represent those of their affiliated organizations, or those of the publisher, the editors and the reviewers. Any product that may be evaluated in this article, or claim that may be made by its manufacturer, is not guaranteed or endorsed by the publisher.
