# Supplementary figures and images for: Methamphetamine induces transcriptional changes in cultured HIV-infected mature monocytes that may contribute to HIV neuropathogenesis
Source: Front Immunol. 2022 Aug 18;13:952183. doi: 10.3389/fimmu.2022.952183 (PMC9433802; doi:10.3389/fimmu.2022.952183)

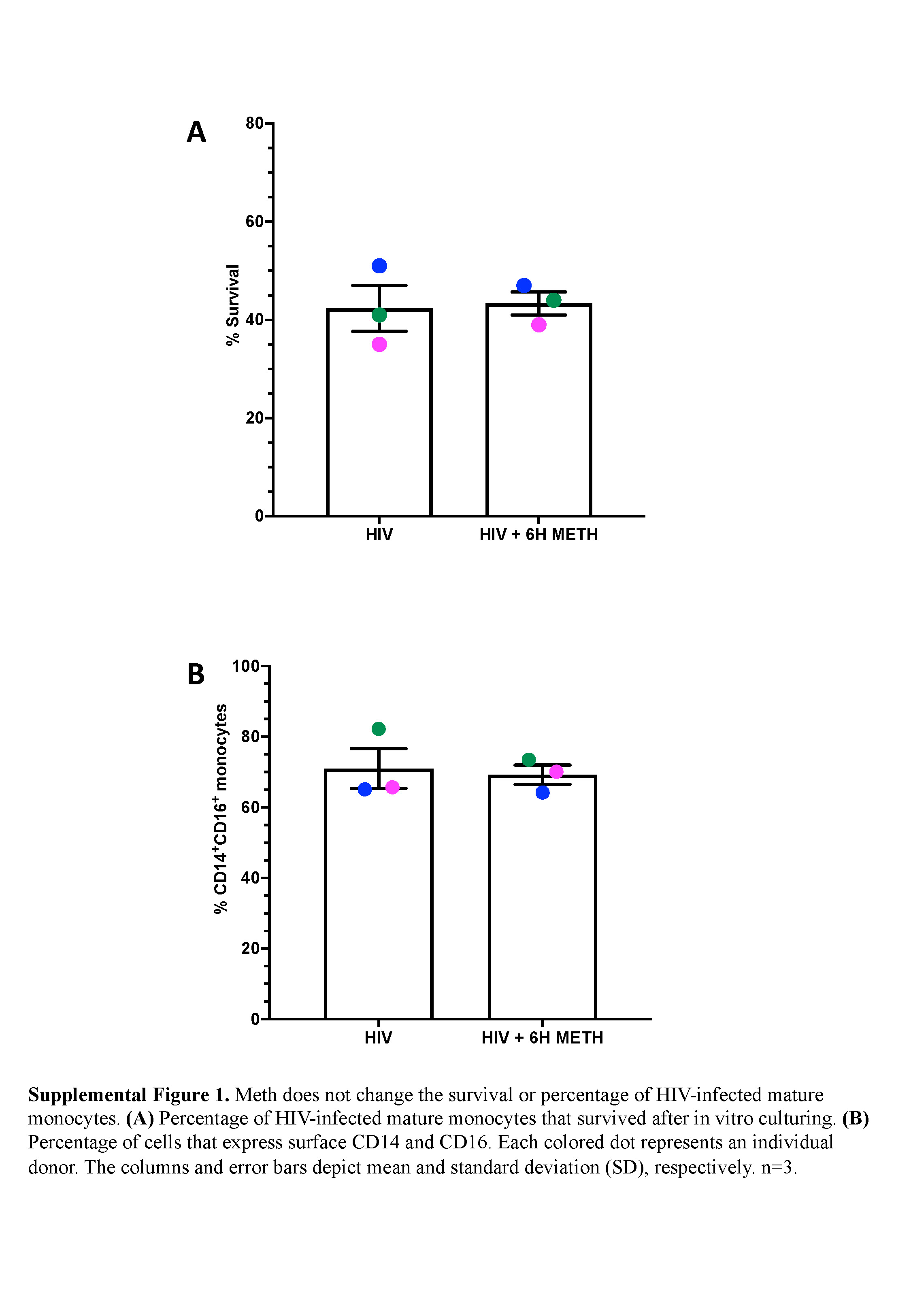

Supplement: Supplementary file 1 [file Image_1.jpeg]

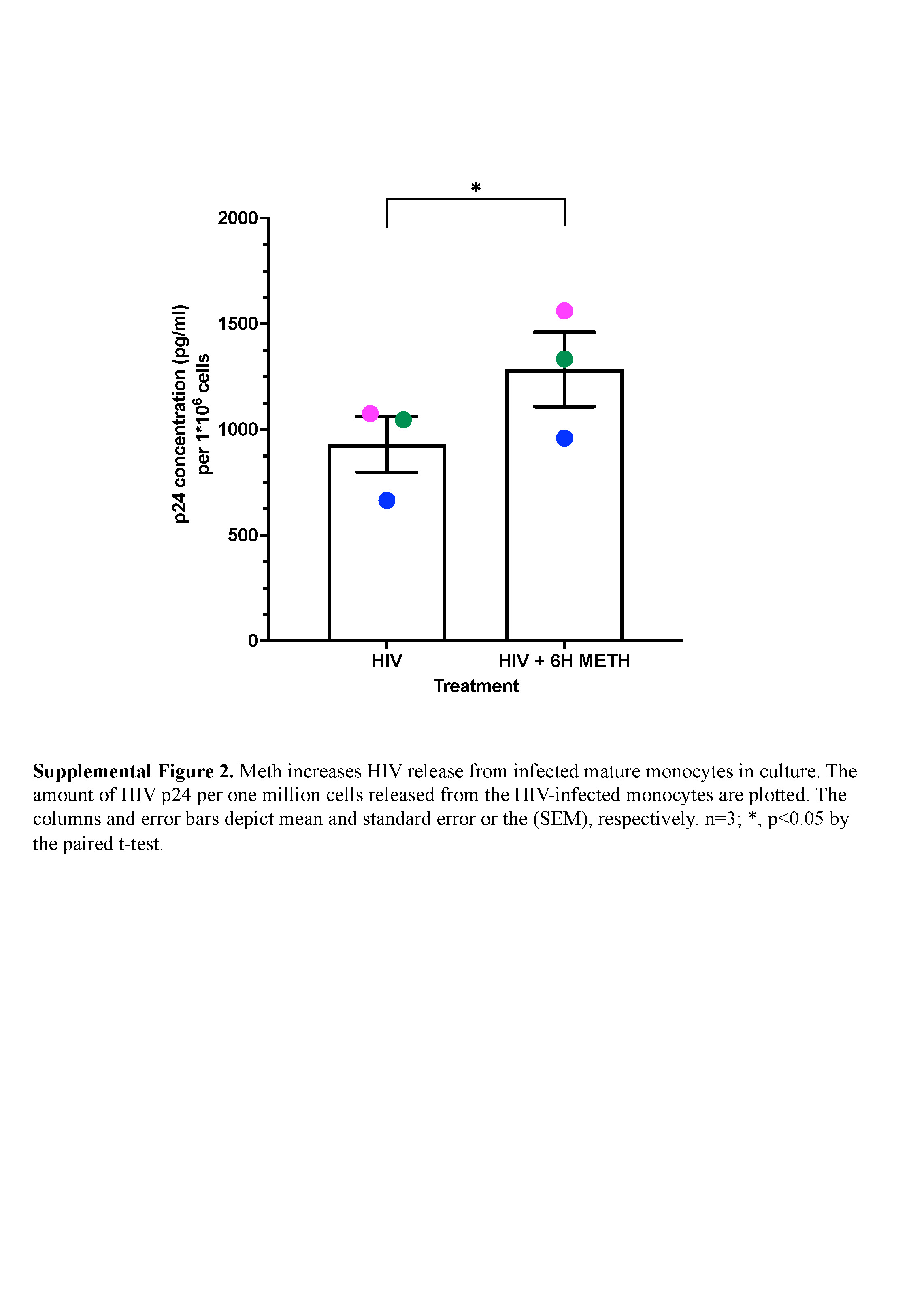

Supplement: Supplementary file 2 [file Image_2.jpeg]
